# Comparing two types of perspective taking as strategies for detecting distress amongst parents of children with cancer: A randomised trial

**DOI:** 10.1371/journal.pone.0175342

**Published:** 2017-04-06

**Authors:** Lucie Gouveia, Annie Janvier, France Dupuis, Michel Duval, Serge Sultan

**Affiliations:** 1 Sainte-Justine University Hospital Center (UHC), Montreal, Québec, Canada; 2 Department of Psychology, University of Montreal, Montreal, Québec, Canada; 3 Department of Pediatrics, University of Montreal, Montreal, Québec, Canada; 4 Faculty of Nursing, University of Montreal, Montreal, Québec, Canada; York University, UNITED KINGDOM

## Abstract

**Objective:**

To compare two perspective taking strategies on (i) clinicians’ ability to accurately identify negative thoughts and feelings of parents of children with cancer, and (ii) clinician distress.

**Methods:**

Sixty-three hematology-oncology professionals and nursing students watched a video featuring parents of children with cancer. Participants were randomly assigned to one of two groups. In the *imagine-self* group, they were instructed to imagine the feelings and life consequences which they would experience if they were in the parents’ position. In the *imagine-other* group, they were instructed to imagine the feelings and life consequences experienced by the parents. Parent-clinician agreement on thoughts/feelings was evaluated (standard stimulus paradigm). Clinician distress was also assessed.

**Results:**

The intervention was effective in manipulating perspective type. The groups did not significantly differ on parent-clinician agreement. Concentrating on personal feelings (imagine-self strategy) did predict lower agreement when controlling for trait empathy. Clinician distress was higher in the imagine-self group.

**Conclusion:**

Although the link between perspective type and detection of distress remains unclear, the results suggest that clinicians who highly focus on their own feelings tend to be less accurate on parental distress and experience more distress themselves.

**Practice implications:**

This research could potentially improve communication training and burnout prevention.

## Introduction

Pediatric cancer is an extremely stressful life event for parents. For many, it is a traumatic experience [1]. Wiener, Kazak, Noll, Patenaude, and Kupst [2] recently published a set of standards recommending that youth with cancer *and* family members ‘routinely receive systematic assessments of their psychosocial health care needs’. Adopting a family-centered approach is especially important in pediatric care, since children and their parents significantly influence each other’s psychosocial adjustment to chronic illness [3, 4]. In addition, addressing parental distress is thought to allow for better parent-clinician collaboration and, ultimately, improved medical care [5].

### Detecting parental distress with empathic skills

Studies clearly show that clinicians struggle to accurately detect distress in adults affected by cancer [6–8], as well as parents of pediatric patients [9]. These findings advocate for teaching simple communication skills to clinicians [10, 11] and using formal screening tools [12, 13]. Unfortunately, very little is known on teachable empathic skills that allow for better recognition of negative emotions.

As objective and user-friendly screening tests may be, they have limited validity and lack the flexibility of relational abilities. Empathic skills could potentially compensate for these shortcomings. In fact, recent studies have reported positive associations between physician empathy and accurate detection of patient distress [14, 15].

One factor that explains the lack of research and training in empathic skills is the breadth of the term “empathy”. For the purposes of this paper, we shall refer to “clinical empathy”, defined by Coulehan et al. [16] as “the ability to understand the […][person’s] situation, perspective, and feelings and to communicate that understanding to the […][person]”. Although empathy is associated with stable personality traits [17], research suggests that it can also be learned [18, 19]. The challenge lies in the identification of simple teachable techniques embedded in the broader skill of clinical empathy. One such technique is empathic communication, a style of interaction which demonstrates compassionate understanding. Two studies have found that clinicians who received extensive training in empathic communication were better at detecting distress in adult cancer patients than those who did not [10, 11].

A second way of teaching empathy is through cognitive skills such as perspective taking. To our knowledge, no study has rigorously examined how perspective taking strategies could enhance detection of distress in adults affected by chronic illness. The present study aims to initiate this line of research by comparing two ways of taking a parent’s perspective.

### Two types of perspective taking

According to Batson [20], there are two types of perspective taking. On one hand, taking an *imagine-self* perspective entails imagining oneself in the other’s situation to infer his/her thoughts and feelings. On the other hand, taking an *imagine-other* perspective involves imagining what the other is thinking and feeling, without necessarily concluding that this person experiences life events in the same way as oneself. The first strategy is associated with relatively more self-oriented distress and behaviour, whereas the second is associated with relatively less personal distress and more altruistic behaviour [21–23]. For instance, Lamm et al. [22] found that participants who had taken an imagine-other perspective, while watching a video of individuals in pain, reported less personal distress (e.g. feeling troubled, low-spirited, alarmed) and more empathic concern (e.g. feeling compassionate, touched, concerned), compared to participants who had adopted an imagine-self perspective.

One group of researchers has examined the relationship between perspective type and accurate detection of distress. Lobchuk and colleagues found that informal caregivers who adopt an imagine-other perspective tend to show less discrepancy with cancer patients on symptom ratings, compared to those who adopt an imagine-self perspective [24–26]. Their studies have important methodological limits, including a poor measure of caregiver accuracy and a theoretical error in the imagine-other instructions. Nonetheless, their findings suggest that the imagine-other perspective could lead to higher accuracy. Batson’s research sheds light on the mechanism(s) involved. One way the imagine-self perspective could evoke self-oriented distress is by blurring the boundary between mental representations of self and other. In support of this idea, neuroimaging studies have found that the imagine-self and imagine-other perspectives show differences in brain activation in areas associated with self-other differentiation [22, 27–29]. Other studies have found that the imagine-self perspective is associated with more self-related thoughts [30] and higher perception of self-other overlap [31]. If the imagine-other perspective does in fact allow for better self-other differentiation than the imagine-self perspective, then it should lead to more accurate perception of the other’s thoughts and feelings. A second explanation could be that personal distress interferes with perception, as suggested by research in emotion recognition [32–34]. Since the imagine-other perspective has been associated with relatively less personal distress and is thought to allow for better self-other differentiation, it seems more appropriate than the imagine-self perspective for detecting distress in parents affected by cancer.

### Objectives

Our first objective was to compare two types of perspective taking on clinicians’ ability to accurately infer parents’ negative thoughts and feelings. We hypothesized that clinicians taking an imagine-other perspective would show higher accuracy than those taking an imagine-self perspective. Our second objective was to verify previous findings linking the imagine-self perspective with relatively more personal distress than the imagine-other perspective [21]. An exploratory objective was to examine whether clinicians’ personal distress could be tested as a possible mediator of the relationship between perspective type and clinician accuracy. The three objectives were successfully addressed in the order described above.

## Methods

### Development of the stimulus video

A stimulus video based on interviews conducted with parents of children with cancer was developed for the study. Five parents were recruited at the Sainte-Justine UHC. To ensure sufficient content, parents with relatively long cancer journeys were selected. Their child needed to be minimum one year post-diagnosis and five years of age or older. Parents needed to be fluent in French. Two-hour filmed interviews were scheduled for parents who accepted to participate. The questions were semi-structured and covered the parent’s cancer journey and emotional experience.

Segments of three interviews were selected for the final video according to ease of communication during the interview and variety of negative thoughts and feelings reported post-interview. To increase ecological validity, parents with different expressivity scores on the Berkeley Expressivity Questionnaire [35, 36] were selected.

Since using the original videos would have biased the results, the selected segments were slightly modified and reproduced by professional actors at the hospital’s medical simulation center. To ensure valid reproductions, the actors studied the recordings, in addition to fully typed scripts. They were given the thoughts and feelings reported by parents at particular time points, and were asked to replicate these parts as close to the original as possible. Each segment was filmed several times to allow for feedback on tone of voice, facial expression, posture, mannerisms, etc. The actors signed a confidentiality form before accessing the material.

The final stimulus video lasts 27 minutes. The first part is composed of six segments, where the parents share parts of their story. The second is composed of 18 segments, where they discuss emotional aspects of their journey.

### Recruitment

Professionals in pediatric hematology-oncology (physicians, nurses, occupational therapists, physiotherapists and social workers) and nursing students were recruited from April to December 2015. Professionals were selected from a registry of 92 healthcare providers at the Charles-Bruneau Cancer Center (Sainte-Justine UHC). The principal investigator sent them an electronic letter describing the study and offering possible participation dates. An announcement containing similar information was posted throughout the department. The nursing students were recruited through the Faculty of Nursing Sciences at the University of Montreal. This was achieved through in-class announcements and distribution of handouts. To be eligible, participants needed to not have collaborated on the project in any way. Professionals needed to have been practicing in hematology-oncology on a regular basis, for a minimum of one year.

### Design overview

A quasi-experimental design with two groups and one post-test evaluation was developed for the present study. After signing consent and confidentiality forms, participants watched the stimulus video. Right before the viewing, each participant received instructions intended to induce one of the two perspective types: imagine-self or imagine-other. Instructions for the imagine-self group read as following: ‘In the following minutes, you will watch interviews featuring three parents of children with cancer. While viewing the video, **try to imagine how you would feel if you were living the same thing as each of these parents and how this would affect your life**’. Alternately, participants in the imagine-other group were instructed to ‘**try to imagine how each parent feels about his/her story and how this has affected their lives**’. Random stratification was employed to form the imagine-self and imagine-other groups on the basis of two lists (nursing students versus hematology-oncology professionals). Participant names were ordered according to scheduled date of participation. For each list, assignment of participants to the imagine-self and imagine-other groups was done in alternate manner. This procedure was done separately for men and women so that the groups could be balanced on gender.

During the second part of the video, participants were reminded of their instructions and parent-clinician agreement on the content of negative thoughts and feelings was evaluated through a self-report method (see Measures). Participants then completed a questionnaire measuring their personal distress. Participation lasted approximately one hour. Additional take-home questionnaires measuring trait empathy were given to them. After returning the questionnaires, participants received a compensation in the form of monetary gifts and donations to parent care services, for a total of 50$ per participant. The participants were also offered free meals.

This protocol was approved by the Sainte-Justine UHC ethics committee (#4016) and the University of Montreal ethics committee for health research.

### Measures

#### Effectiveness of perspective taking manipulation

Effectiveness of the experimental manipulation was evaluated post-intervention, as in Batson’s initial study [21]. For assessment of the imagine-self perspective, participants were asked to rate how much they had concentrated on their own feelings, as if they were personally experiencing the stories told by the parents. For assessment of the imagine-other perspective, participants rated how much they had concentrated on the parents’ feelings. Both answers were rated on scales ranging from 0 (not at all) to 10 (very).

#### Parent-clinician agreement on negative thoughts and feelings

Parent-clinician agreement on the parents’ thoughts and feelings was measured using the *standard stimulus paradigm of empathic accuracy* [37]. Post-interview, the parents had been asked to watch their video and record every thought and feeling they remembered having. This was done using a standardised thought/feeling recording form adapted from the original version used in Marangoni et al. [37]. Each time the parent remembered having had a thought or feeling, they were to stop the tape and record the time, the specific content of the thought/feeling, and its valence (positive, neutral, or negative). Instructions on the form are meant to minimize biases due to self-censorship.

When showing the final video to the participants, the research assistant stopped it at specific time points where the parents had reported thoughts or feelings. The participants then had to infer the content of each thought/feeling and record it on another standardized thought/feeling inference form [37]. For the present study, the tape was stopped at 18 feelings or thoughts, 15 of which were negative in valence.

As in the standard paradigm [37], four independent raters (trained psychology students blind to group assignment) rated the extent to which each inference was similar to the actual thought/feeling reported by the parent. The rating scale ranged from 0 to 2, where 0 = ‘essentially different content’, 1 = ‘similar, but not the same, content’, and 2 = ‘essentially the same content’ [38]. Scores for each inference were averaged across raters. A mean value of .75 was obtained for inter-rater reliability, suggesting a substantial level of agreement [39]. The total parent-clinician agreement score was computed by dividing the total sum of inference scores by the maximum points, multiplied by 100.

#### Personal distress

Post-intervention, participants completed the Emotional Response Scale [21, 40]. The test measures personal distress and empathic concern in reaction to a person in need/distress. Of particular interest for this study is the Personal Distress subscale, composed of eight items/emotions (alarmed, grieved, troubled, distressed, upset, disturbed, worried, and perturbed) rated on a scale of 1 to 7. Principal components analyses support the two-factor structure of this questionnaire [41]. In this study, Cronbach alphas of .80 and .84 were obtained for empathic concern and personal distress, respectively.

#### Clinician empathic traits

Participants completed the Interpersonal Reactivity Index [42, 43], a measure of individual differences in empathy composed of four factors: Empathic Concern, Perspective Taking, Personal Distress and Fantasy. The subscales have demonstrated good internal consistency, with Cronbach alphas ranging from .70 to .78 [44]. They have also demonstrated good construct validity [42, 45, 46].

### Statistical analyses

In preliminary analyses, the effectiveness of the perspective taking manipulation was examined. To determine whether relative levels of concentration on personal feelings and parent feelings varied as a function of experimental group, a repeated measures factorial ANOVA was performed with experimental group (imagine-other vs. imagine-self) as the between-group factor, type of feelings attended to (personal feelings vs. parent feelings) as the repeated measure, and level of concentration as the dependent variable.

For the first objective, an independent samples t-test was performed to compare the experimental groups on parent-clinician agreement on negative thoughts/feelings. Clinician empathic traits were added as control variables in ANCOVAs and in additional correlation analyses.

For the second objective, the groups were compared on personal distress in an independent samples t-test.

Previous studies comparing the emotional responses associated with the two types of perspective taking have reported moderate to large effect sizes [21, 22]. Power analyses [47] indicated that a sample of about 60 participants would achieve 80% power for a Cohen’s *d* of .60 on our first objective, representing a between-group difference of 10% on parent-clinician agreement.

Analyses for both studies were performed through IBM SPSS Statistics 20 and an alpha level of .05 was set for statistical significance.

## Results

**Fig 1:**
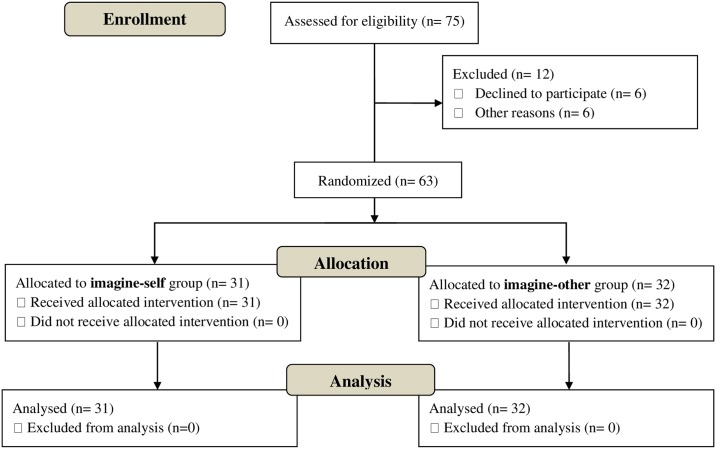
Participant flow chart following consolidated standards of reporting trials guidelines.

Seventy-five participants were assessed for eligibility (Fig 1). Six refused to participate. Six additional participants dropped out before the beginning of the study for reasons related to time constraints and/or lack of motivation. The difference between these individuals and those who participated are unknown. A total of 29 hematology-oncology professionals and 34 nursing students participated in this study. Out of the 63 participants, 53 (84.1%) were female and 50 (79.4%) were white. Most were either nurses or nursing students (77.8%; see Table 1 for sample description).

**Table 1:** Sample description.

Variables	n (%)	M	SD
Age		30.59	11.03
Gender			
Men	10 (15.9)		
Women	53 (84.1)		
Ethnicity			
White	50 (79.4)		
African	5 (7.9)		
Haitian	3 (4.8)		
Asian	2 (3.2)		
South American	2 (3.2)		
Arab	1 (1.6)		
Profession			
Nurse	21 (33.3)		
Nursing student ^a^	28 (44.4)		
Medical Doctor	7 (11.1)		
Occupational Therapist	3 (4.8)		
Physiotherapist	3 (4.8)		
Social Worker	1 (1.6)		
Experience in oncology ^b^		3.80	5.98
Experience with parents ^b^		6.13	9.06

^a^ Six nursing students already possessed a technical degree in nursing.

^b^ Experience is represented in years.

### Effectiveness of perspective taking manipulation

**Fig 2:**
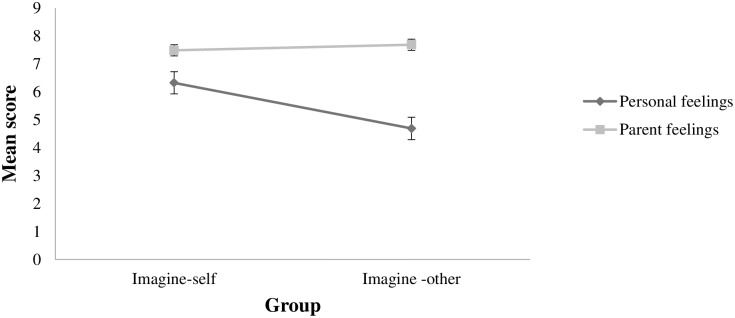
Clinicians' level of concentration on personal and parent feelings as a function of experimental group (p < .01). Error bars represent standard deviations.

In the repeated measures ANOVA, a significant interaction effect was found for group and type of feeling, F(1, 61) = 9.27, *p* < .01, with a medium effect size (partial *η*^*2*^ = .132; Fig 2). Tukey analyses showed a significant difference in clinicians’ level of concentration on personal feelings between the imagine-self group (*M* = 6.32, SD = .40) and the imagine-other-group (*M* = 4.69, SD = .40), *p* < .05. The groups did not significantly differ on level of concentration on parent feelings.

### Main outcomes

#### Parent-clinician agreement on negative thoughts and feelings

No significant difference was found between the imagine-self group (*M* = 53.20; SD = 8.70) and the imagine-other group (*M* = 56.32; SD = 8.76) on parent-clinician agreement on negative thoughts and feelings, *t*(61) = -1.41, *p* = n.s. Similar non-significant results were found when controlling for the effect of empathic traits in ANCOVAs.

In correlational analyses performed across groups, concentration on personal feelings was associated with lower parent-clinician agreement when controlling for three of the four empathic traits. Moderate partial correlation values of *r*(55) = -.29, -.27, and -.27 (*p* < .05) were obtained when controlling for Empathic Concern, Personal Distress, and Fantasy, respectively. The partial correlation obtained when controlling for Perspective Taking was slightly lower, r(55) = -.22, *p* = n.s.

#### Clinician personal distress

**Fig 3:**
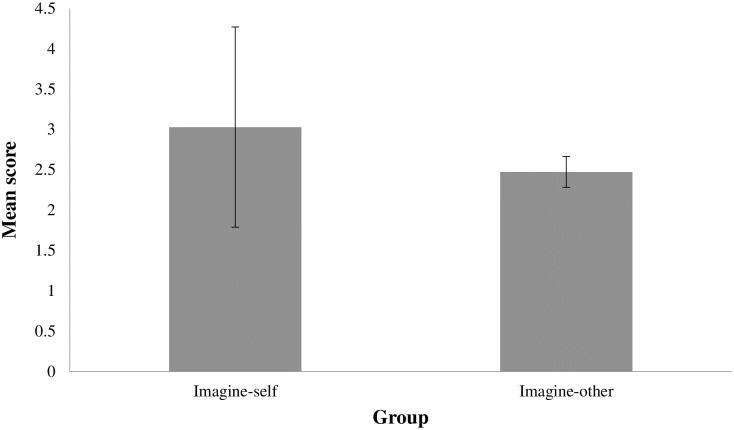
Mean level comparison of clinician personal distress between the two experimental groups (p < .05). Error bars represent standard deviations.

The average level of clinician personal distress was significantly higher in the imagine-self group (*M* = 3.03, SD = 1.24) than in the imagine-other group (*M* = 2.47; SD = .19), *t*(55) = 2.02, *p* < .05 (Fig 3), with a large difference (*d* = .625). Note that since Levene’s test indicated unequal variances (F = 4.23, p = .044), degrees of freedom were adjusted from 61 to 55.

No significant difference on clinician empathic concern was found between the imagine-self group (*M* = 4.15, SD = 1.30) and the imagine-other group (*M* = 4.01, SD = 1.32), *t*(61) = .45, *p* = n.s.

In additional analyses performed across groups, concentration on personal feelings and personal distress were found to be significantly correlated at a moderate level, *r*(61) = .25, *p* < .05.

The full dataset used for this study is available as supporting information (S1 File).

## Discussion

The present study compared two types of perspective taking as clinical strategies for detecting distress in parents of children with cancer. Supporting the experimental manipulation’s effectiveness, clinicians instructed to adopt an imagine-self perspective reported higher levels of concentration on personal feelings compared to clinicians instructed to adopt an imagine-other perspective. In contrast to our predictions, the imagine-self group did not show significantly lower agreement with parents on their negative thoughts and feelings. However, correlational analyses showed a negative association between parent-clinician agreement and level of concentration on personal feelings (imagine-self strategy). Replicating previous findings, clinicians in the imagine-self group reported higher levels of personal distress.

Our main hypothesis stipulated that clinicians instructed to adopt an imagine-other perspective would show higher agreement with parents on their thoughts and feelings, compared to clinicians instructed to adopt an imagine-self perspective. Although this was not supported by the analyses, we estimated that only seven more participants would have been needed to obtain a significant difference with the same effect size. Moreover, the intervention *was* successful at manipulating the degree to which clinicians concentrated on their personal feelings, which in turn predicted lower agreement when controlling for trait empathy. Since concentrating on personal feelings is an imagine-self strategy, this finding suggests a certain link between the imagine-self perspective and poorer accuracy. Still, it is important to examine why these correlational results did not translate into a significant group difference. Perhaps the intervention was insufficiently effective. The extent to which clinicians concentrated on their personal feelings may have largely depended on their own preferences, in addition to experimental manipulation. For the purpose of replication, the present study uses Batson’s methodology in its simple form. However, future studies could increase the effectiveness of the intervention by using a procedure that constantly reminds participants of their instructions. For example, thought/feeling reporting forms could be slightly altered. The imagine-self form could read ‘If I were in that person’s situation, I imagine that I would think/feel…’ before each inference. Alternately, the imagine-other form could read ‘I imagine that this parent thinks/feels…’ We would expect this to considerably increase the chances of finding a significant group difference in parent-clinician agreement. Experimental studies with larger sample sizes and more elaborate interventions may eventually indicate a causal relationship between perspective type and clinician accuracy.

Previous findings on the emotional outcomes of perspective taking were replicated by the present study [21, 22]. As predicted, clinicians in the imagine-self group reported more personal distress than did those in the imagine-other group. The greater amount of variance in the imagine-self group could be explained by possible conflicts between the imagine-self instructions and clinicians’ preferences. When asked which strategy they most frequently used to infer parents’ experience, 83% reported usually relying on observed parent characteristics as compared to 27% usually relying on their own emotions. While some studies have linked clinical empathy with compassion fatigue and burnout, others have reported positive associations with psychological well-being and work satisfaction [48–50]. The distinct emotional reactions associated with the imagine-self and imagine-other perspectives provide a potential explanation for these discrepant findings. Perhaps empathy does lead to excessive personal distress when a clinician relies too heavily on an imagine-self perspective. Alternately, an imagine-other perspective may play a protective role against distress, without compromising accuracy or empathic concern. This idea resembles the common distinction between sympathy and empathy [51, 52]. Similar to previous findings [20, 31], no significant group difference was found on empathic concern. This finding suggests that complete emotional detachment is not necessary for protecting clinicians from detrimental psychological distress.

In contrast to our predictions, personal distress showed no correlation with parent-clinician agreement. These results do not suggest that personal distress could mediate the hypothesized link between perspective type and parent-clinician agreement. Perhaps a more plausible mediator is self-other differentiation. In this case, poor differentiation, rather than high personal distress, would explain why clinicians who were more focused on their personal feelings (imagine-self perspective) tended to obtain lower parent-clinician agreement scores. As stated earlier, the two types of perspective taking show neurological and cognitive differences associated with self-other differentiation [22, 27–31]. A relatively feasible goal for future studies would be to examine whether the activation of specific brain areas mediates the relationship between perspective type and parent clinician-agreement.

Concluding that the imagine-self perspective is a categorically useless strategy would be unempirical. According to Batson, imagining oneself in another’s shoes (i.e. the imagine-self perspective) most likely acts as a useful “stepping stone” to understanding the other’s personal experience with limited information [20]. However, previous findings suggest that self-other differentiation is important, at least as a subsequent step in the empathic process. Such oscillation between shared and differentiated representations might be the optimal perspective taking strategy. Although the imagine-other instructions allow for this oscillation, they do not specify it. In future studies, it would be pertinent to have a third group of participants imagine the other as being both similar and dissimilar to them. This approach corroborates Carl Rogers’ conceptualisation of empathy which he defined as ‘the perception of the internal frame of reference of another with accuracy and with the emotional components and meanings which pertain thereto as if one were the person, but without ever losing the "as if" condition’ [53].

### Study limitations

There are a few limits to this protocol. First, the effectiveness scales used in the present study have yet to be formally validated. These were selected for the purpose of replicating Batson’s original protocol. To our knowledge, no other measures of imagine-self and imagine-other perspective taking have been developed. Future investigations could avoid such a limitation by relying more heavily on experimental methods. Alternately, perspective type could be inferred through a mixed methods approach. In the paradigm proposed by Davis et al. (30), participants retrospectively record the thoughts they had while watching the video, which are then coded as self- or other-related. Such a method could be used to examine how clinician thought content is related to parent-clinician agreement. Secondly, clinicians who chose to participate were most probably more interested in empathy compared to those who did not. Since motivation is an important part of empathy, the results may not be representative of health care providers as a whole. Thirdly, one could criticize the stimulus video’s validity, given that the actual parents are not featured in it. However, any effect caused by differences between the two versions was controlled for, as the same video was shown to all participants. Moreover, such an effect would most likely cause a conservative ceiling effect, since the major challenge for the actors was moderating the expression of emotions they intended to convey. Failing at this would facilitate the task. This may have happened in the present study, and could partially explain the lack of between-group difference on our main outcome. Finally, many participants reported difficulty in generating words to describe the feelings they perceived in the parents. However, offering a list of words might render the task too easy [54]. To deal with this challenge, a little extra time was allowed for participants to finish writing their answers.

### Conclusion

With the application of fundamental socio-cognitive theory and methodology in the oncology setting, the present study contributes important findings on the clinical utility of two perspective taking strategies for detecting distress in parents of children with cancer, as well as other persons affected by cancer. Although parent-clinician agreement on parental distress was not found to significantly differ between experimental groups, the results suggest that the imagine-other perspective is a generally more adaptive clinical tool than the imagine-self perspective. Being associated with higher personal distress, the imagine-self perspective may place clinicians at greater risk for professional burnout. In addition, the imagine-self perspective was not found to yield any advantages for detection of distress. On the contrary, it was associated with poorer parent-clinician agreement when measured as level of concentration on personal feelings. The findings point to self-other differentiation as a plausible key strategy for enhancing clinician accuracy and psychological well-being.

### Practice implications

This research helps broaden the repertoire of trainable empathic skills and strategies for detecting distress. A recent multi-center survey conducted in the UK indicates that professionals in cancer care are eager to learn more about emotion recognition and management as part of psychological training and support [55]. The present study suggests that emotion identification could possibly be improved by practicing shared attention between self and other as separate mental representations.

## Supporting information

S1 File Full dataset. (DOCX)

S2 File Dataset codebook. (DOCX)

## References

[1] Ljungman L, Hovén E, Ljungman G, Cernvall M, von Essen L. Does time heal all wounds? A longitudinal study of the development of posttraumatic stress symptoms in parents of survivors of childhood cancer and bereaved parents. Psycho-Oncology. 2015;24(12):1792–8. 10.1002/pon.3856 26042579 PMC5033007

[2] Wiener L, Kazak AE, Noll RB, Patenaude AF, Kupst MJ. Standards for psychosocial care for children with cancer and their families: An introduction to the special issue. Pediatr Blood Cancer. 2015;S5(62):S419–24. 10.1002/pbc.25675 PMC6397048 26397836

[3] Sultan S, Leclair T, Rondeau É, Burns W, Abate C. A systematic review on factors and consequences of parental distress as related to childhood cancer. European Journal of Cancer Care. 2015. 10.1111/ecc.12361 PMC5049674 26354003

[4] Mullins LL, Molzon ES, Suorsa KI, Tackett AP, Pai ALH, Chaney JM. Models of resilience: Developing psychosocial interventions for parents of children with chronic health conditions. Family Relations. 2015;64(1):176–89.

[5] Spinetta JJ, Masera G, Eden T, Oppenheim D, Martins AG, van Dongen-Melman J, et al Refusal, non-compliance, and abandonment of treatment in children and adolescents with cancer. A report of the SIOP Working Committee on Phychosocial Issues in Pediatric Oncology. Medical and Pediatric Oncology. 2002;38(2):114–7. 11813177 10.1002/mpo.1283

[6] Chidambaram S, Deshields T, Potter P, Olsen S, Chen L. Patient and provider concordance on symptoms during the oncology outpatient clinic visit. Journal of Community and Supportive Oncology. 2014;12(10):370–7. 10.12788/jcso.0080 25853259

[7] Keller M, Sommerfeldt S, Fischer C, Knight L, Riesbeck M, Löwe B, et al Recognition of distress and psychiatric morbidity in cancer patients: a multi-method approach. Annals of Oncology. 2004;15(8):1243–9. 10.1093/annonc/mdh318 15277265

[8] Werner A, Stenner C, Schüz J. Patient versus clinician symptom reporting: how accurate is the detection of distress in the oncologic after-care? Psycho-Oncology. 2011;21(8):818–26. 10.1002/pon.1975 21544897

[9] Patel SK, Mullins W, Turk A, Dekel N, Kinjo C, Sato JK. Distress screening, rater agreement, and services in pediatric oncology. Psycho-Oncology. 2011;20(12):1324–33. 10.1002/pon.1859 20925136

[10] Merckaert I, Libert Y, Delvaux N, Marchal S, Boniver J, Etienne A-M, et al Factors influencing physicians' detection of cancer patients' and relatives' distress: Can a communication skills training program improve physicians' detection? Psycho-Oncology. 2008;17(3):260–9. 10.1002/pon.1233 17575569

[11] Fukui S, Ogawa K, Ohtsuka M, Fukui N. Effect of communication skills training on nurses' detection of patients' distress and related factors after cancer diagnosis: A randomized study. Psycho-Oncology. 2009;18(11):1156–64. 10.1002/pon.1429 19194993

[12] Haverman L, van Oers HA, Limperg PF, Houtzager BA, Huisman J, Darlington A-S, et al Development and validation of the distress thermometer for parents of a chronically ill child. The Journal of Pediatrics. 2013;163(4):1140–6. 10.1016/j.jpeds.2013.06.011 23910979

[13] Leclair T, Carret A-S, Samson Y, Sultan S. Stability and repeatability of the Distress Thermometer (DT) and the Edmonton Symptom Assessment System-Revised (ESAS-r) with parents of childhood cancer survivors. PLoS ONE. 2016;11(7):e0159773 10.1371/journal.pone.0159773 27454432 PMC4959708

[14] Gouveia L, Lelorain S, Brédart A, Dolbeault S, Bonnaud-Antignac A, Cousson-Gélie F, et al Oncologists’ perception of depressive symptoms in patients with advanced cancer: accuracy and relational correlates. BMC Psychology. 2015;3(6). 10.1186/s40359-015-0063-6 PMC4359512 25815195

[15] Yagil D, Biron M, Pat D, Mizrahi-Reuveni M, Zoller L. Accurate diagnosis of patients' distress levels: The effect of family physicians' ability to take the patients' perspective. Patient Education and Counseling. 2015;98(12):1631–5. 10.1016/j.pec.2015.07.008 26215572

[16] Coulehan JL, Platt FW, Egener B, Frankel R, Lin C-T, Lown B, et al “Let me see if I have this right …”: Words that help build empathy. Annals of Internal Medicine. 2001;135(3):221–7. 11487497 10.7326/0003-4819-135-3-200108070-00022

[17] del Barrio V, Aluja A, García LF. Bryant's empathy index for children and adolescents: Psychometric properties in the Spanish language. Psychological Reports. 2004;95(1):257–62. 10.2466/pr0.95.1.257-262 15460381

[18] Bonvicini KA, Perlin MJ, Bylund CL, Carroll G, Rouse RA, Goldstein MG. Impact of communication training on physician expression of empathy in patient encounters. Patient Education and Counseling. 2009;75(1):3–10. 10.1016/j.pec.2008.09.007 19081704

[19] Pehrson C, Banerjee SC, Manna R, Shen MJ, Hammonds S, Coyle N, et al Responding empathically to patients: Development, implementation, and evaluation of a communication skills training module for oncology nurses. Patient Education and Counseling. 2016;99(4):610–6. 10.1016/j.pec.2015.11.021 26686992 PMC4962546

[20] Batson CD. Two forms of perspective taking: Imagining how another feels and imagining how you would feel In: Keith DM, William MPK, Julie AS, editors. Handbook of imagination and mental simulation. New York, NY: Psychology Press; US; 2009 p. 267–79.

[21] Batson CD, Early S, Salvarani G. Perspective taking: Imagining how another feels versus imagining how you would feel. Personality and Social Psychology Bulletin. 1997;23(7):751–8.

[22] Lamm C, Batson CD, Decety J. The Neural Substrate of Human Empathy: Effects of Perspective-taking and Cognitive Appraisal. Journal of Cognitive Neuroscience. 2007;19(1):42–58. 10.1162/jocn.2007.19.1.42 17214562

[23] Batson CD, Lishner DA, Carpenter A, Dulin L, Harjusola-Webb S, Stocks EL, et al “… As you would have them do unto you”: Does imagining yourself in the other's place stimulate moral action? Personality and Social Psychology Bulletin. 2003;29(9):1190–201. 10.1177/0146167203254600 15189613

[24] Lobchuk MM, Vorauer JD. Family caregiver perspective-taking and accuracy in estimating cancer patient symptom experiences. Social Science & Medicine. 2003;57(12):2379–84. 14572844 10.1016/s0277-9536(03)00132-1

[25] Lobchuk MM, Degner LF, Chateau D, Hewitt D. Promoting enhanced patient and family caregiver congruence on lung cancer symptom experiences. Oncology Nursing Forum. 2006;33(2):273–82. 10.1188/06.ONF.273-282 16518443

[26] Lobchuk MM, McClement SE, Daeninck PJ, Shay C, Elands H. Asking the right question of informal caregivers about patient symptom experiences: Multiple proxy perspectives and reducing interrater gap. Journal of Pain and Symptom Management. 2007;33(2):130–45. 10.1016/j.jpainsymman.2006.07.015 17280919

[27] Ruby P, Decety J. Effect of subjective perspective taking during simulation of action: a PET investigation of agency. Nat Neurosci. 2001;4(5):546–50. 10.1038/87510 11319565

[28] Ruby P, Decety J. How would you feel versus how do you think she would feel? A Neuroimaging Study of Perspective-Taking with Social Emotions. Journal of Cognitive Neuroscience. 2004;16(6):988–99. 10.1162/0898929041502661 15298786

[29] Jackson PL, Brunet E, Meltzoff AN, Decety J. Empathy examined through the neural mechanisms involved in imagining how I feel versus how you feel pain. Neuropsychologia. 2006;44(5):752–61. 10.1016/j.neuropsychologia.2005.07.015 16140345

[30] Davis MH, Soderlund T, Cole J, Gadol E, Kute M, Myers M, et al Cognitions associated with attempts to empathize: How do we imagine the perspective of another? Personality and Social Psychology Bulletin. 2004;30(12):1625–35. 10.1177/0146167204271183 15536244

[31] Myers MW, Laurent SM, Hodges SD. Perspective taking instructions and self-other overlap: Different motives for helping. Motivation and Emotion. 2014;38(2):224–34.

[32] Gery I, Miljkovitch R, Berthoz S, Soussignan R. Empathy and recognition of facial expressions of emotion in sex offenders, non-sex offenders and normal controls. Psychiatry Research. 2009;165(3):252–62. 10.1016/j.psychres.2007.11.006 19167095

[33] Lampic C, von Essen L, Peterson VW, Larsson G, Sjoden P-O. Anxiety and depression in hospitalized patients with cancer: Agreement in patient-staff dyads. Cancer Nursing. 1996;19(6):419–28. 8972974 10.1097/00002820-199612000-00002

[34] Schmid P, Schmid Mast M. Mood effects on emotion recognition. Motivation and Emotion. 2010;34(3):288–92.

[35] Gross JJ, John OP. Facets of emotional Expressivity: Three self-report factors and their correlates. Personality and Individual Differences. 1995;19(4):555–68.

[36] Gross JJ, John OP. Berkeley Expressivity Questionnaire. Measurement Instrument Database for the Social Science. 2013.

[37] Marangoni C, Garcia S, Ickes W, Teng G. Empathic accuracy in a clinically relevant setting. Journal of Personality and Social Psychology. 1995;68(5):854–69. 7776183 10.1037//0022-3514.68.5.854

[38] Ickes W, Hodges S. Empathic Accuracy in Close Relationships In: Simpson J, Campbell L, editors. Handbook of Close Relationships. Oxford: Oxford University Press; 2013 p. 348–7.

[39] Landis JR, Koch GG. The Measurement of Observer Agreement for Categorical Data. Biometrics. 1977;33(1):159–74. 843571

[40] Coke JS, Batson CD, McDavis K. Empathic mediation of helping: A two-stage model. Journal of Personality and Social Psychology. 1978;36(7):752–66.

[41] Batson CD, Fultz J, Schoenrade PA. Distress and empathy: Two qualitatively distinct vicarious emotions with different motivational consequences. Journal of Personality. 1987;55(1):19–39. 3572705 10.1111/j.1467-6494.1987.tb00426.x

[42] Davis MH. Measuring individual differences in empathy: Evidence for a multidimensional approach. Journal of Personality and Social Psychology. 1983;44(1):113–26.

[43] Guttman H, Laporte L. Alexithymia, empathy, and psychological symptoms in a family context. Comprehensive Psychiatry. 2002;43(6):448–55. 10.1053/comp.2002.35905 12439832

[44] Davis MH. A Multidimensional Approach to Individual Differences in Empathy. JSAS Catalog of Selected Documents in Psychology. 1980;10:85.

[45] Lawrence EJ, Shaw P, Baker D, Baron-Cohen S, David AS. Measuring empathy: reliability and validity of the Empathy Quotient. Psychological Medicine. 2004;34(05):911–20. 15500311 10.1017/s0033291703001624

[46] Yarnold PR, Bryant FB, Nightingale SD, Martin GJ. Assessing physician empathy using the interpersonal reactivity index: A measurement model and cross-sectional analysis. Psychology, Health & Medicine. 1996;1(2):207–21.

[47] Faul F, Erdfelder E, Albert-Georg L, Buchner A. G*Power 3: A flexible statistical power analysis program for the social, behavioral, and biomedical sciences. Behavior Research Methods. 2007;39(2):175–91. 17695343 10.3758/bf03193146

[48] Picard J, Catu-Pinault A, Boujut E, Botella M, Jaury P, Zenasni F. Burnout, empathy and their relationships: a qualitative study with residents in General Medicine. Psychology, Health & Medicine. 2016;21(3):354–61. 10.1080/13548506.2015.1054407 26075525

[49] Lamothe M, Boujut E, Zenasni F, Sultan S. To be or not to be empathic: the combined role of empathic concern and perspective taking in understanding burnout in general practice. BMC Family Practice. 2014;15(1):1–7. 24456299 10.1186/1471-2296-15-15 PMC3914722

[50] Zenasni F, Boujut É, Woerner A, Sultan S. Burnout and empathy: three hypotheses. British Journal of General Practice. 2012;62(600):346–7. 10.3399/bjgp12X652193 22781970 PMC3381244

[51] Hojat M, Spandorfer J, Louis DZ, Gonnella JS. Empathic and sympathetic orientations toward patient care: Conceptualization, measurement, and psychometrics. Academic Medicine. 2011;86(8):989–95. 10.1097/ACM.0b013e31822203d8 21694570

[52] Nightingale SD, Yarnold PR, Greenberg MS. Sympathy, empathy, and physician resource utilization. J Gen Intern Med. 1991;6(5):420–3. 1744756

[53] Rogers CR. A theory of therapy, personality and interpersonal relationships as developed in the client-centered framework In: Koch S, editor. Psychology: a study of a science III. Formulations of the person and the social context. New York: McGraw Hill; 1959 p. 184–256.

[54] Ickes W. Empathic Accuracy. Journal of Personality. 1993;61(4):587–610.

[55] Laffan AJ, Daniels J, Osborn M. Profiling the psychological training and support needs of oncology staff, and evaluating the effectiveness of a Level 2 Psychological Support Training Programme workshop. Journal of Psychosocial Oncology. 2015:686–702. 10.1080/07347332.2015.1082170 26317638

