# Supplementary material for: Comparing two types of perspective taking as strategies for detecting distress amongst parents of children with cancer: A randomised trial
Source: PLoS One. 2017 Apr 6;12(4):e0175342. doi: 10.1371/journal.pone.0175342 (PMC5383290; doi:10.1371/journal.pone.0175342)
Supplement: S2 File — (DOCX) [file pone.0175342.s002.docx]

**Dataset Codebook**

| Variable name | Meaning |
| --- | --- |
| ID | Participant identity number |
| Group | Experimental condition (1 = Imagine-self; 2 = Imagine-other) |
| Gender | Participant gender (1 = female; 2 = male) |
| Children | Whether the participant has children (1 = yes; 2 = no) |
| Age | Participant age |
| Ethnicity | Participant ethnicity (1 = White; 2 = Asian; 3 = South American; 4 = Haitian; 5 = African; 6 = Arab) |
| Title | Professional title (1 = Pivot nurse; 2 = other type of nurse; 3 = Physician; 4 = student; 5 = Social worker; 6 = Physiotherapist; 7 = Occupational therapist; 8 = Nurse and student) |
| Oncology | Work experience in oncology (months) |
| Experience_parents | Work experience with parents (months) |
| Length_consultation_patient | Average duration of consultations with patients (minutes) |
| Frequency_patient | Average number of consultations per patient in a week |
| Length_consultation_parent | Average duration of consultations with parents (minutes) |
| Frequency_parent | Average number of consultations per parent (or couple) in a week |
| Emotions | Whether the patients discuss emotions with their patients’ parents (1 = yes; 2 = no) |
| Difficult | Number of difficult encounters in the past week |
| Satisfying | Number of satisfying encounters in the past week |
| Self_efficacy | Feeling of competency in detecting emotional distress in parents or adult patients |
| Conc_self_intervention | Level of concentration on personal emotions during the experimental intervention (1 – 10) |
| Conc_other_intervention | Level of concentration on the parents’ emotions during the experimental intervention (1 – 10) |
| Habit | Strategy most frequently used for understanding parents’ emotions on a daily basis (1 = observe parents’ emotions; 2 = use own emotions) |
| Strategy_intervention | Strategy most used by participant during experimental intervention (1 = observe parents’ emotions; 2 = use own emotions) |
| ERS | Emotional response scale. Each item rated on a scale of 1 to 7. |
| Emp_Con | ERS Empathic Concern factor |
| Pers_Dis | ERS Personal Distress factor |
| IRI | Interpersonal Reactivity Index. Each item rated on a scale of 1 to 5. |
| IRI_R | IRI reverse-coded item |
| IRI_Pers_Tak | IRI Perspective Taking factor |
| IRI_Fant | IRI Fantasy factor |
| IRI_Emp_Con | IRI Empathic Concern factor |
| IRI_Pers_Dis | IRI Personal Distress factor |
| Agreement | Standardized scores for level of agreement with parents on thoughts and feelings (0-100) |
